# Analyzing Arabic Handwriting Style through Hand Kinematics

**DOI:** 10.3390/s24196357

**Published:** 2024-09-30

**Authors:** Vahan Babushkin, Haneen Alsuradi, Muhamed Osman Al-Khalil, Mohamad Eid

**Affiliations:** 1 Applied Interactive Multimedia Lab, Engineering Division, New York University Abu Dhabi, Abu Dhabi P.O. Box 129188, United Arab Emirates; 2 Tandon School of Engineering, New York University, New York, NY 11201, USA; 3 Arabic Studies Program, New York University Abu Dhabi, Abu Dhabi P.O. Box 129188, United Arab Emirates

**Keywords:** handwriting, handwriting style, deep learning, temporal convolutional networks, sensorimotor learning, machine learning

## Abstract

Handwriting style is an important aspect affecting the quality of handwriting. Adhering to one style is crucial for languages that follow cursive orthography and possess multiple handwriting styles, such as Arabic. The majority of available studies analyze Arabic handwriting style from static documents, focusing only on pure styles. In this study, we analyze handwriting samples with mixed styles, pure styles (Ruq’ah and Naskh), and samples without a specific style from dynamic features of the stylus and hand kinematics. We propose a model for classifying handwritten samples into four classes based on adherence to style. The stylus and hand kinematics data were collected from 50 participants who were writing an Arabic text containing all 28 letters and covering most Arabic orthography. The parameter search was conducted to find the best hyperparameters for the model, the optimal sliding window length, and the overlap. The proposed model for style classification achieves an accuracy of 88%. The explainability analysis with Shapley values revealed that hand speed, pressure, and pen slant are among the top 12 important features, with other features contributing nearly equally to style classification. Finally, we explore which features are important for Arabic handwriting style detection.

## 1. Introduction

Handwriting is a complex human sensorimotor skill that entails the integration and simultaneous coordination of human visual-perceptual, cognitive, and motor systems [1]. To produce readable handwriting, individuals rely on visual and haptic feedback to coordinate the movement of the hand, arm, and fingers. Mastering the skill of handwriting is an important milestone in child development—legible and aesthetic handwriting has a direct influence on the the educational process, academic success, and self-confidence [2]. The integration of artificial intelligence with other technological advancements opens new possibilities for deployment of tablets, touch devices, and hand tracking for analysis of factors influencing the quality of handwriting in healthy adults during handwriting evaluation [3], detection/prediction of handwriting difficulties [4], and novel remediation/intervention methods [5]. These factors include legibility, speed, pen grip, pressure, handwriting movements, style, and error corrections [6]. Understanding the role these factors play in handwriting is important for developing innovative approaches for handwriting development and evaluation [7] as well as for rehabilitation [5].

The Arabic script is the third most commonly used writing system in the world by the number of users (after Latin and Chinese scripts) and the second by the number of countries [8]. It follows the Abjad system, which is a right-to-left writing system; it is cursive by nature; it is sensitive to the context and uses ligatures to connect letters; and it possesses multiple writing styles [9,10]. Arabic is also known for polymorphism: the shape of the letter changes relative to its position in the word (e.g., separate, starting, middle, and ending) [11]. Furthermore, Arabic has letters of similar shape but of different sounds, which are distinguished by the dots over and under the letters. Single, double, and combined diacritical marks are placed under or over the Arabic letter to facilitate pronunciation of unwritten vowels. These marks can be placed or omitted depending on the writer [11]. Finally, aesthetics play a crucial role in expressing individual and cultural identities in the Arab world by enhancing the script’s legibility and beauty while conveying cultural meanings and values that reflect the aesthetic traditions and historical contexts of the Arabic script [9,10,12]. All of these unique features of Arabic orthography enable addressing a diverse variety of handwriting skills, including understanding which features influence handwriting style.

Handwriting style refers to the characteristic way in which written characters are formed and connected within a specific script. Styles serve practical and aesthetic purposes, influencing readability, speed of writing, and cultural conventions associated with written communication [13]. Handwriting style is an important component of handwriting proficiency, as the consistent adherence to one of the handwriting styles contributes to readability and aesthetics of handwriting [14]. Additionally, legibility is significantly influenced by how characters are drawn, which is largely determined by personal writing style, since the ambiguously formed characters can lead to poor legibility of the handwritten sample [14,15]. Depending on the script type, handwriting styles can vary significantly in complexity and features. In general, for western scripts, the handwriting style determines whether the text has been written in printed (manuscript) text, cursive text, or a mixture of the two. However, it is not the case for cursive languages as Arabic, which is known for multiple writing styles; the most common ones are Nasta’liq, Naskh, Kufi, Ruq’ah, Thuluth, and Diwani [9]. Similar to western scripts, the styles of Arabic can be mixed. In western scripts, children and adults usually mix styles; however, studies show that this does not affect the legibility of handwriting [16]. Furthermore, handwriting speed is also affected by handwriting style. Those students who employed a mixed style of handwriting demonstrated higher handwriting speeds compared to those who exclusively used only manuscript or only cursive styles [16]. Additionally, a mixed-cursive style handwriting is usually evaluated highly in terms of legibility compared to handwriting that follows only one style [16].

Detecting handwriting style has several advantages. In education, incorporating a style categorization module in personalized handwriting acquisition will help students to develop a consistent adherence to one style at the advanced stages of learning Arabic handwriting. Currently available personalized handwriting acquisition systems categorize several aspects of handwriting, such as character difficulty, errors, and time spent on the task, to evaluate the quality of handwriting [17]. Style categorization can be also used as an additional indicator for evaluating the quality of handwriting [17]. The most recent studies employ handwriting to diagnose cognitive impairment in adults by categorizing video images of different handwriting tasks performed on a Wacom tablet into either 12 classes (depending on the type of the video, e.g., pentagon/pentagon error) or two classes (healthy/not healthy), depending on the presence or absence of cognitive disorder [18]. Alterations in handwriting might be a sign of Alzheimer’s and Parkinson’s disease as well as of mild cognitive impairments (MCIs) [19,20] that could potentially lead to style inconsistency. Detection of inconsistency or mixture of styles in individuals who used to adhere one of the styles before might indicate development of neurocognitive problems. Therefore, automatic style classification can potentially be employed in diagnostic and rehabilitation systems. Style inconsistency might also indicate emotional changes, and the style classification model that is able to detect mixed or inconsistent handwriting styles can be applied for emotion detection [21]. Finally, style classification is actively used for authorship detection of ancient documents, helping to identify the origin and date of the manuscript [22], which potentially can be applied in individual authentication systems.

There are limited studies on style analysis and classification of Arabic handwriting. Most of the works have focused on style classification from static text documents. For example, in [23], the authors recognize six styles (Diwani, Kufi, Naskh, Farsi, Ruq’ah, and Thuluth) of ancient Arabic manuscripts by segmenting letters and applying Gabor filters (GFs) and local binary pattern (LBP) to extract features for further processing with the support vector machine (SVM) classifier, achieving 86.84% accuracy. Similarly, in [24], the authors deploy texture descriptors to recognize Diwani, Kufi, Naskh, Farsi, Ruq’ah, Thuluth, Maghribi, Mohakik, and square-Kufi styles, achieving the best performance of 94.7% with the binarized statistical image feature (BSIF) descriptor with the SVM classifier. In [22], the authors classified the Naskh and Ruq’ah styles from ancient manuscripts using SIFT and SURF algorithms for feature extraction and processed these features with Gaussian Naïve Bayes (GNB), decision tree (DT), random forest (RF), and k-nearest neighbor (KNN) classifiers, achieving a maximum accuracy of 92% with the GNB classifier. Deep learning approaches, such as a pre-trained MobileNet convolutional neural network, were also used for style classification of handwritten Arabic manuscripts, achieving an accuracy of 95.8% [25]. The most recent study [26] used transfer learning for classifying nine different Arabic calligraphy styles and achieved an accuracy of 91%, recall of 89%, precision of 92%, and F1-score of 90%.

However, there are no studies that use the dynamic features collected from the stylus and hand kinematics to classify the style. The closest to style classifications from stylus and hand kinematics are studies focusing on online Arabic handwriting recognition. Online handwriting recognition involves automatic conversion of a text written on the surface of an electronic device (tablet) that captures the *x* and *y* coordinates of the stylus tip, pen-up/pen-down movements, pen tilt, and applied force. The captured stylus kinematics embed the description of contextual data like position, direction, and velocity [27]. For example, in [28], the authors developed a method for online Arabic character recognition using Kohonen neural networks with elliptic Fourier coefficients of the x(t) and y(t) components of pen positions as input features, achieving an 88.38% recognition rate for both clearly and roughly written characters. Similarly, Drotár et al. used x(t) and y(t) components of pen position, pen pressure, azimuth, and altitude, as well as a variable indicating if the pen tip was touching the surface, to engineer spatiotemporal and kinematic features for dysgraphia detection with 80% accuracy [4]. Another example is a study by Tlemsani et al. [29] that deployed a time delay neural network (TDNN) for online handwriting recognition of Arabic characters using stylus x(t) and y(t) coordinates, direction, curvature, and pen up/down states as features, achieving up to 99.61% recognition rates. To the best of our knowledge, one of the few papers that has explored alternative modalities to tablets is by Alemayoh et al. [30], in which the authors analyzed readings from inertial measurement units (IMUs) and from three force sensors embedded in a pen to recognize 36 handwritten alphanumeric characters with an accuracy of 99.05%. To the best of our knowledge, there are no studies that use real-time style detection based on the kinematics features of Arabic handwriting.

In this work, we propose a system for style detection of Arabic handwriting from stylus and hand kinematics, focusing on two Arabic handwriting styles: Naskh and Ruq’ah. The Naskh style, derived from the Thuluth style, evolved into its distinct form during the 10th century. It is characterized by its simplicity and high legibility, especially in smaller font sizes, with thin, round lines. Naskh has become the most popular style for Arabic book publishing, particularly for the Holy Quran [22]. In contrast, Ruq’ah, introduced in the 9th century, is the simplest style for everyday, non-official handwriting. Its round and fluid style [31], combined with its simplicity, has made Ruq’ah the preferred choice for daily writing in the eastern Arab world. This style features dense ligature structures, a thick baseline, and short horizontal strokes [32]. In comparison with other studies where static text is used, we utilize both stylus and hand kinematics features to classify Ruq’ah and Naskh styles. Furthermore, while previous studies, such as [23,24], have primarily focused on detecting pure handwriting styles, our study not only addresses the detection of pure Ruq’ah and Naskh styles but also includes the classification of handwriting samples with mixed and inconsistent styles. We also utilize interpretability of the developed model to highlight prominent features pertaining to Arabic handwriting styles. To the best of our knowledge, there have been no previous attempts to classify handwriting samples of mixed and inconsistent styles.

### 1.1. Experiment Setup

**Figure 1:**
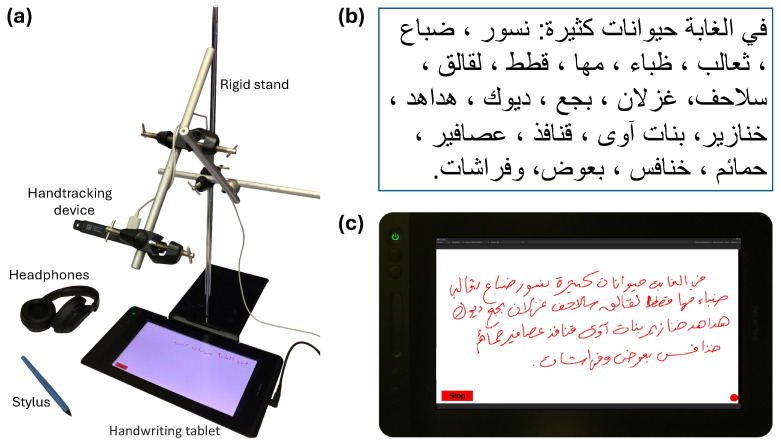
(**a**) Experiment setup, (**b**) sample text dictated to subjects, (**c**) user interface with a sample of subject’s handwriting (text is shown using the Naskh style).

The experiment setup shown in Figure 1a consists of a Huion GT-116 (Huion, Shenzhen, China) tablet paired with a pen-like stylus and an Ultraleap Stereo IR 170 hand motion tracker (Ultraleap, Bristol, UK), which is placed on the rigid stand. The system is flexible for relocation and adjustments.

### 1.2. Participants

Fifty native Arabic speaking participants were recruited for this study, all older than 18, with no history of neuromuscular or orthopedic dysfunction or dysgraphia, who attended school with Arabic instruction language from grade 1, and who predominantly use their right hand. Participants were required to be available for in-person meeting (for recording the handwriting tasks). The study was conducted in compliance with the Declaration of Helsinki, following its norms and regulations, and with a protocol authorized by the New York University Abu Dhabi Institutional Review Board (IRB: #HRPP-2023-93).

### 1.3. Experimental Task and Protocol

The text dictated to subjects shown in Figure 1b contained 26 Arabic words that easily fit the available space of the tablet screen. The text included all 28 letters of the Arabic alphabet, incorporating all essential connectivity positions of Arabic orthography. Unique glyphs (such as ك) in the text were represented in all their connected/unconnected forms. However, homoglyphs such as ب, ت, ث were not exhaustively represented individually in all their connected/unconnected forms but only as a group, given the similarity between these letters.

Before the experiment began, participants listened to the entire text sample at a speed of 20 words per minute. Then, participants were asked to press the “Start” button on the tablet screen (Figure 1c) to initiate the data recording. The button label changed to “Stop”, inviting subjects to stop the recording at the end of the dictation. The text was dictated through headphones, with the dictation speed adjusted to the writing speed of the subject. At the end of the dictation session, the subject was instructed to press the “Stop” button to submit the recorded data. The data submitted consisted of tablet screenshots (Figure 1c), seven stylus-kinematic features recorded from the tablet, and 110 hand-kinematic features recorded by the hand tracking device. The subject was asked to repeat the handwriting task six times, each time with the same text. In total, the data for 303 handwritten samples were collected from 50 participants. Some participants required an additional round of training due to unfamiliarity with writing on the tablet surface. The high quality of these training data led to their inclusion in the final dataset.

### 1.4. Expert Evaluation and Measures

**Figure 2:**
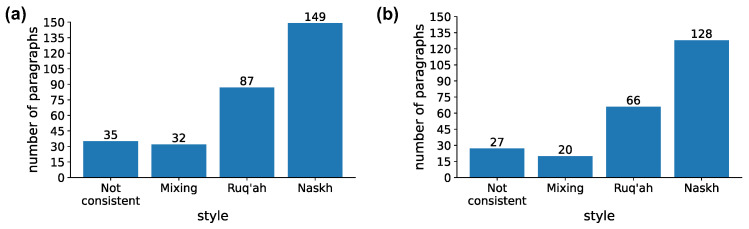
Distribution of the expert’s evaluation of style consistency by paragraphs. (**a**) Original distribution, (**b**) after retaining paragraphs that correspond to the prevailing style of the subject.

An Arabic teaching expert was recruited to assess the style consistency of the screenshots of handwriting samples. The expert evaluated the style consistency of the handwriting sample, assessing whether it was not consistent, mixed, or consistent. Additionally, for samples evaluated as consistent, the expert was asked to determine the handwriting style of the sample, either Ruq’ah or Naskh. The inclusion criteria for the expert were: (1) more than 10 years of experience in teaching Arabic handwriting and (2) currently working in official (statutory work) or extra-official settings (non-statutory work). Figure 2a shows the distribution of style consistency evaluations. In general, there are 241 paragraphs out of 303 with consistent styles, out of which 128 are written in the Naskh style and 66 in Ruq’ah.

### 1.5. Data Preparation

In this study, paragraphs that correspond to the majority of a subject’s style were considered. In the event there was an equal number of paragraphs with different styles for the same subject, the style of the paragraphs to be included in the dataset was determined randomly. If an equal number of paragraphs exhibit mixed or inconsistent styles compared to those with pure styles, preference was given to the paragraphs with mixed or inconsistent styles. The rationale for this decision is grounded in the assumption that if the subject’s written paragraphs exhibit an equal or predominant amount of inconsistent or mixed styles compared to pure styles, it suggests that the subject’s overall style leans more towards inconsistency or mixing of styles. Otherwise, only paragraphs that constituted the majority of style for the given subject were included. Following this strategy, a total number of 241 paragraphs were retained out of the 303 paragraphs. The final distribution of paragraphs by four styles remains similar to the original (see Figure 2b).

### 1.6. Features

A total of 117 kinematic features were recorded; 7 stylus features were recorded through the tablet and 110 hand kinematics features from the hand tracking device (see Table 1). The data were recorded with a sampling rate of 25 Hz and synchronized to a unified time stamp.

### 1.7. Model Architecture

**Figure 3:**
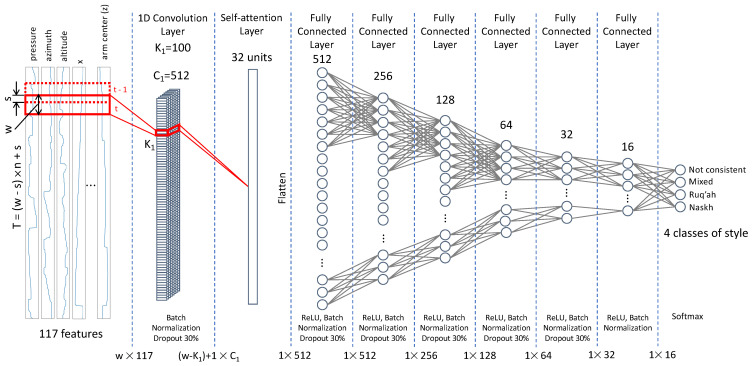
The proposed architecture with two temporal convolution layers: *w*—the length of the window, *s*—overlap, *n*—number of windows, *T*—length of the entire paragraph, $K_{1}$—kernel sizes of the first/second 1D-CNN layers, $C_{1}$—number of channels in first/second 1D-CNN layers.

The proposed model illustrated in Figure 3 is based on the Temporal Convolutional Network (TCN) architecture [33], which utilizes 1D convolutions to extract features encoded over time [34]. TCNs can update layers’ weights simultaneously at each time step, offering superior performance over Long Short-Term Memory networks for long time series data [34,35]. TCNs can handle sequences of any length, preventing information leakage from future to past events [36]. However, TCNs can struggle with capturing dependencies between long-range patterns due to the limited receptive field of the convolutional kernels [34]. Adding a self-attention layer to TCNs enhances their ability to capture these long-range dependencies [37] and infer hidden associations in features, enabling the network to learn more complex and irregular patterns [38], further making the model’s performance more interpretable [37].

The model (Figure 3) consists of one TCN layer represented by a one-dimensional convolutional layer (1D-CNN). The use of one TCN layer is based on the assumption that the first layer extracts temporal dependencies. Visualization of feature maps after the first 1D-CNN layer confirmed this assumption. Adding another 1D-CNN layer results in minimal improvement in the model’s accuracy. The TCN layer is followed by a self-attention layer that processes the hidden representation and extracts a global temporal attention mask. The learning occurs within the subsequent four fully connected layers. The number of fully connected layers and the number of neurons in each layer are determined empirically, starting with the simplest possible architecture.

The input layer takes a matrix of 117 features and *t* time points and feeds it to the first convolution layer with $C_{1}=512$ channels. Due to the variable length *T* of an original paragraph, the sliding window approach was used to create more samples. Thus, sliding a window of length *w* with overlap *s* along a paragraph of length *T* splits it into *n* samples of size w×117, such as T=(w−s)×n+s, where w=960 time points—an optimal window length found through the hyperparameter search. The convolution is performed by sliding a kernel of size $K_{1}=100$ along the time dimension of each window. The resulting $\left(w-K_{1}\right)+1\times C_{1}$ matrix is passed to the input of the self-attention layer of 32 units, then flattened and passed to the fully connected layers with 512, 256, 128, 64, 32, 16, and finally 4 neurons, equal to the number of classes (Not Consistent, Mixing, Naskh, or Ruq’ah). Batch normalization and a dropout of 30% were applied after convolutional and fully connected layers to stabilize the learning process and prevent overfitting. The Rectified Linear Unit (ReLU) activation function was used in all layers except the final output layer, which uses Softmax. The model was trained over 500 epochs using the AdaDelta optimizer [39] and categorical cross-entropy loss. Due to the limited sample size, the learning rate could not be adjusted with callbacks; instead, the optimal learning rate of $10^{-3}$ was found empirically and remained constant during training. To address class imbalance, an oversampling method was applied to the training data before using them to train the network.

### 1.8. Shapley Values

Shapley values, a concept from game theory [40], are used to evaluate each feature’s impact on model predictions [41,42]. They are computed by iteratively substituting each feature with uniformly-distributed random values and retraining the model. Shapley values are determined by comparing the model’s predictions with a random feature to those with the original feature across all validation instances, then averaged across the validation set to assess each feature’s overall influence. Formally, the Shapley value of a feature $f\in F=\left\{1,\cdots ,d\right\}$ from the set of all feature indices *F* is a weighted average of its marginal contributions $M_{f}\left(S\right)$. Each marginal contribution $M_{f}\left(S\right)$ represents the difference in evaluation after introducing the feature of index *f* to a sub-model S⊂F, i.e., $M_{f}\left(S\right)=C\left(S\cup \left\{f\right\}\right)-C\left(S\right)$, where *C* is an evaluation function.

The formula to calculate a Shapley value ($\phi_{f}$) for a feature index is:

(1) $$\phi_{f}=\sum_{S\in 2^{F\setminus \left\{f\right\}}}\omega \left(S\right)M_{f}\left(S\right),$$

where $\omega \left(S\right)=\frac{|S|!\left(|F|-|S|-1\right)!}{\left|F\right|}!$ are the weights [42].

### 1.9. Optimal Parameter Search

Handwriting style is typically assessed based on the entire sample rather than individual letters or words. Due to the limited sample size (191 paragraphs) and imbalanced distribution of samples of Naskh and Ruq’ah styles, a sliding window method is used to increase the sample size for model training and evaluation. The window must be large enough to include enough words to represent the overall style of the paragraph accurately.

The configuration of the minimum network architecture (one TCN layer, one self-attention layer, and the output layer) was determined by conducting a grid search to find the optimum number of output channels of the TCN layer, as well as the size of the convolutional kernel. The search for the number of output channels was performed over a set of 8 channel values, log-scaled with a base of from $2^{3}=8$ to $2^{10}=1024$, using the median window size of 896. As for the kernel size, a set of 13 kernel sizes were tested ranging from 3 to 800. Note that the upper limit of the kernel sizes is specified by the length of shortest sample (1774). The model for each configuration was run for 100 epochs. With the optimal kernel size of 100 and 521 channels, the network with minimal architecture was able to achieve overall accuracy of 62.6%. Further experiments demonstrated that these parameters allow us to achieve the highest accuracy/precision/recall/F1-score values).

**Figure 4:**
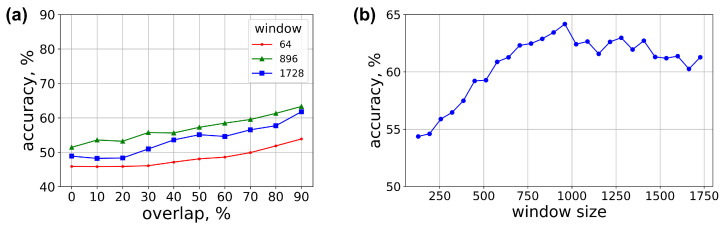
Searching for optimal (**a**) overlap and (**b**) window size.

To determine the optimal time window length, a grid search was conducted across 27 different window lengths. Initially, the best overlap size was found by training the model with three fixed window lengths: 128 (smallest), 896 (median), and 1728 (largest, close to the shortest sample length of 1774 and a multiple of 64). Overlaps ranged from 0% to 90% in 10% steps. The step size of 64 was chosen based on the minimum possible word length. Average metrics (accuracy, precision, recall and F1-score) were calculated across 5 folds and 5 runs for each window length, selecting a different random seed for each run. Results showed a linear increase in metrics values with higher overlap percentages, leading to the adoption of a 90% overlap (see Figure 4a).

A parameter search was conducted to determine the optimal window length for assigning a legibility score to text samples. Window lengths ranged from 128 to 1728 with steps of 64 and an overlap of 90%, where the choice of step 64 is dictated by the minimum possible word length. For each iteration, the model was trained and validated over 5 folds, repeated 5 times with different random seeds, and performance metrics such as accuracy, precision, recall and F1-score were averaged over all folds for each run and then across all 5 runs. As shown in Figure 4b, all metrics increased from a window length of 128, achieving a maximum value at 960 and gradually decreasing until 1728. At a window length of 960, accuracy reached the maximum value. Thus, an optimal window length of 960 allows us to generate a larger number of samples.

## 2. Results

### 2.1. Model Performance Evaluation

**Figure 5:**
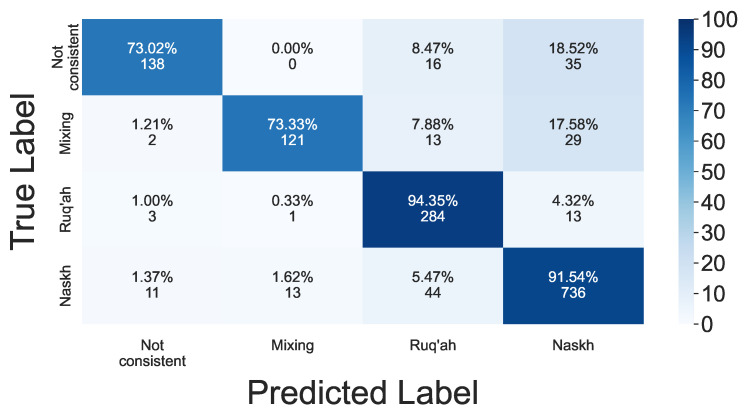
Average of confusion matrices over 5 folds. The diagonal shows average recall across 5 folds for each of the 4 classes.

The proposed model is evaluated in terms of accuracy, precision, recall, and F1-score using 5-fold cross-validation. All stylus and hand kinematics features were used. Data leakage was prevented by forming folds at the paragraph level. Also, an additional stratification across subjects was conducted to ensure all subjects are represented by their paragraphs both in training and testing datasets by moving randomly selected paragraphs of a given subject from the training to the testing set if that subject was underrepresented in the testing set. For instance, if, after the fold assignment, there were four paragraphs of a given subject in the training set and no paragraphs in the testing set, then one of that subject’s paragraphs randomly selected from the training set was moved to the testing set. The paragraphs were split with a sliding window of length 960 with 90% overlap, with the number of samples in the testing set ranging between 1257 to 1284 within the 5 folds. The average accuracy of the model was 88%, with average recall of 83%, average precision of 88%, and average F1-score of 85%. The average accuracy for predicting the Ruq’ah style was 88%, average precision was 80%, average recall 94%, and average F1-score 86%. Similarly, the average accuracy for predicting the Naskh style was 88%, average precision was 91%, average recall 91%, and average F1-score 91%. The confusion matrix averaged over 5 folds is shown in Figure 5.

### 2.2. Feature Analysis

The contribution of each feature to the style prediction was estimated by calculating Shapley values. The model with optimal parameters was 5-fold cross-validated, and Shapley values were evaluated for each model from each fold on 1200 samples of size w×117 obtained from the sliding window approach and randomly selected from the testing set in the given fold. It is recommended to use the testing set for Shapley value calculations because it allows for the evaluation of the features’ impact on the model’s generalization performance.

**Figure 6:**
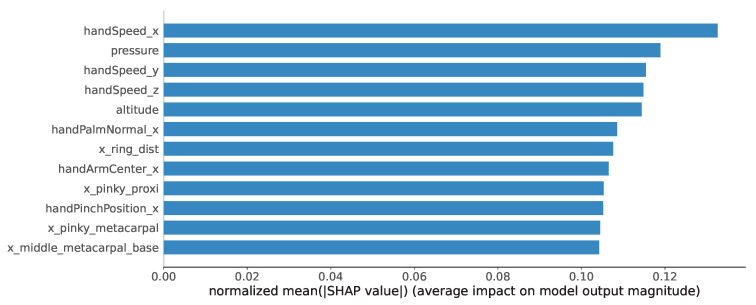
Average of normalized Shapley values across 5 folds.

Only the Shapley values of correctly predicted instances are considered, thus focusing on the features that are influential to valid predictions. For every fold, the calculated Shapley values are first averaged across 960 time points and then over 1200 test instances of size w×117 obtained from the sliding window approach. Finally, the Shapley values for each fold were normalized and averaged to determine which features are prominent on average across folds. The top 12 most prominent features for predicting the style are depicted in Figure 6, showing the normalized Shapley values averaged across folds.

The *x* component of hand speed appears to be prominent across all 5 folds, while the second most prominent feature is pressure. The x,y components of hand speed and stylus altitude appear to be less important and similar by magnitude. The magnitudes of other features decrease gradually but remain very close to each other, which suggests equal contribution of other features towards style prediction.

## 3. Discussion

The proposed model allows us to detect handwriting style with high accuracy. The high recalls of the Naskh and Ruq’ah styles can be explained by significant imbalance in samples with consistent styles (Naskh and Ruq’ah) over samples with mixed styles, as well as the samples that do not follow any style. From the confusion matrix in Figure 5, it is easy to see that, in some cases, the model misclassifies inconsistent and mixed styles as Naskh and Ruq’ah. This is explained not only by the prevalence of Naskh style samples over others but also by the difficulty in distinguishing between samples with pure styles and samples with mixed styles. This is a hard task even for experts, both due to the absence of clear guidelines on which sample can be considered mixed or pure style, and also because even proficient writers can sometimes mix styles. The first circumstance might lead to a biased estimation of style consistency by the expert, depending on their educational and cultural backgrounds. The aim of the model is to eliminate the expert-related bias in style classification tasks.

The focus of this study is to utilize interpretable machine learning techniques, such as Shapley values, to understand which features are the most important for style classification. The model developed for explainability analysis was evaluated with 5-fold cross-validation on the folds formed at the paragraph level. Since the adherence to one of the handwriting styles or to a mixing style is a completely personalized characteristic of handwriting, forming the folds at the subject level (the leave-one-subject-out approach) will deprive the model of capturing the personalized features of those subjects that were “left-out”, i.e., in the testing folds. In particular, it is important for subjects whose fine sensorimotor skills related to handwriting, such as pen-grasp patterns, are different. Hiding personalized features of the same subjects from the model will deteriorate the results of the explainability analysis. Therefore, forming folds at the paragraph level is important for ensuring the model learns all the individual characteristics affecting the handwriting style of each subject.

For explainability analysis, it is crucial to maintain a balance between simplicity and performance in the network architecture. A simpler model enhances interpretability and facilitates the calculation of Shapley values, making it easier to understand the contributions of individual features. However, the model must still perform at an acceptable level to ensure the reliability and relevance of the results. This balance ensures that the model remains both interpretable and effective, allowing for meaningful insights without sacrificing accuracy. In general, deep neural networks outperform shallow architectures by learning and representing more complex functions through hierarchical feature learning, where higher-level abstractions are captured in additional layers. Deep networks are able to model intricate relationships, including long-term dependencies, and are more generalizable. Techniques such as batch normalization and dropout further enhance their robustness by mitigating issues such as vanishing and exploding gradients. To find an optimal model, a search of the architecture was conducted starting from a simple one-layered network with a goal of achieving an accuracy exceeding 85%. The simple architectures resulted in lower accuracies—e.g., an architecture that consisted of one 1D-CNN layer, one self-attention layer, and one output layer achieved the highest accuracy of about 62.6%—which is infeasible for conducting the interpretability analysis. Another obstacle that shallow networks face is class imbalance. Increasing the depth of the learning unit helped mitigate these problems. The architecture proposed in Figure 3 is optimal in terms of depth, accuracy, and computational costs.

The Shapley values calculated for each fold during 5-fold cross-validation suggest that handwriting speed is the dominant feature that is consistent across folds. The importance of other features varies across folds. Interestingly, after the first few top features, the magnitude of Shapley values becomes quite similar, suggesting an equal impact of most of the features on the model performance. The average of normalized Shapley values across folds places the three components of hand speed along with pressure and altitude among the top five important features for style detection. The magnitudes of the Shapley values of these features are prominent and change more sharply than the magnitudes of other features. According to Grahan et al. [16], style can influence handwriting speed—students who mix manuscript and cursive styles were writing faster and with better legibility compared to those who adhere to one style only (either manuscript or cursive). Therefore, hand speed components are expected to be among the top five features.

The parameter search demonstrated that the model needs larger windows to form a conclusion about the style. From Figure 4b, it is clear that model accuracy grows with the window size until it reaches the optimal size of 960. While the smaller windows lead to an increase in the number of samples, they do not contain enough words to make a definite conclusion about the style. On the other hand, larger model windows lead to a drop in accuracy, which can be explained both by increased probability of mixing styles in larger excerpts from text as well as by the model’s overfitting due to the increase in information per window and decrease in the number of samples.

While there is no research on the relationship between handwriting style and applied force, Harris et al. [43] reported an increase in applied force when subjects increased or decreased their usual handwriting speed. Since mixing of handwriting styles is associated with faster speeds [16], it is possible that the mixed style is associated with higher applied forces, i.e., pressure. In this sense, pressure is an important feature for handwriting style prediction.

This study has following limitations: the sample size of 50 subjects is relatively small, and all participants are from an academic environment, thus more accustomed to handwriting than the average person. Most subjects (30) were 18–25 years old, with females outnumbering males two to one. Additionally, hand tracking issues occurred, especially among females, due to hand shape and the use of sunscreen, which interfered with the infrared camera. Future work will analyze kinematic features affecting handwriting style using larger and more diverse samples. The influence of demographic factors such as age and gender will be investigated. Alternative data acquisition systems such as Wacom Bamboo or reMarkable, which allow writing on physical or simulated paper, will be considered for collecting handwriting samples.

## 4. Conclusions

This study focuses on understanding the contribution of stylus and hand kinematics features to classification of Arabic handwriting styles. We propose a Temporal Convolution Network (TCN)-based model capable of classifying samples of pure styles such as Naskh and Ruq’ah as well as mixed samples and those without distinct styles. The model allows us, on average, to achieve accuracy of 88% for four-class classification, with 94% and 91% recall for Ruq’ah and Naskh style classification, respectively. The explainability analysis with Shapley values was conducted to understand which features are most critical in classifying handwriting styles. The Shapley values averaged across 5 folds revealed that hand speed, pressure, and the slant of the pen relative to the writing surface contribute to the model’s decision-making process. However, the magnitudes of Shapley values for other features do not change significantly, suggesting the equal contribution of these features to the model’s ability to distinguish between handwriting styles. The potential applications of the proposed model can be found in personalized handwriting acquisition systems, diagnostics and rehabilitation, emotion detection, and authorship verification.

## Figures and Tables

**Table 1:** The 117 hand and stylus kinematics features.

Feature Type	Feature Name
Stylus kinematics	$\left(x,y,z\right)$ coordinates of the tool tip’s trajectory (z=const), pressure, azimuth, altitude, proximity
Hand kinematics	Fingers: the distal end $\left(x,y,z\right)$ coordinates of the bone for distal, intermediate, and proximal phalanges for the index, middle, ring, and little fingers and distal and proximal phalanges for the thumb, $\left(x,y,z\right)$ coordinates of palm center, $\left(x,y,z\right)$ coordinates of hand pinch position (thumb and index if they are pinched), $\left(x,y,z\right)$ coordinates of hand predicted pinch position, $\left(x,y,z\right)$ coordinates of hand wrist position, $\left(x,y,z\right)$ coordinates of elbow position (estimated if not in view), $\left(x,y,z\right)$ coordinates of hand arm center (midpoint of the bone), $\left(v_{x},v_{y},v_{z}\right)$ palm speed, $\left(n_{x},n_{y},n_{z}\right)$ hand palm normal, $\left(r_{x},r_{y},r_{z},r_{w}\right)$ hand rotation, Palm width, pitch, yaw, roll, Hand pinch strength, hand pinch distance, Hand grab angle, Hand arm length (length of the bone), Hand arm width (average width of flesh around the bone).

## Data Availability

The data are available upon reasonable request sent to the corresponding author.

## References

[1] Bonney M.A. (1992). Understanding and assessing handwriting difficulty: Perspectives from the literature. Aust. Occup. Ther. J..

[2] Chang S.H., Yu N.Y. (2013). Handwriting movement analyses comparing first and second graders with normal or dysgraphic characteristics. Res. Dev. Disabil..

[3] Rosenblum S., Parush S., Weiss P.L. (2003). The in air phenomenon: Temporal and spatial correlates of the handwriting process. Percept. Mot. Skills.

[4] Drotár P., Dobeš M. (2020). Dysgraphia detection through machine learning. Sci. Rep..

[5] Fancher L.A., Priestley-Hopkins D.A., Jeffries L.M. (2018). Handwriting acquisition and intervention: A systematic review. J. Occup. Ther. Sch. Early Interv..

[6] van Drempt N., McCluskey A., Lannin N.A. (2011). A review of factors that influence adult handwriting performance. Aust. Occup. Ther. J..

[7] Maor D., Currie J., Drewry R. (2011). The effectiveness of assistive technologies for children with special needs: A review of research-based studies. Eur. J. Spec. Needs Educ..

[8] Ghali N. (2009). Write It in Arabic: A Workbook and Step-by-Step Guide to Writing the Arabic Alphabet.

[9] Naz S., Hayat K., Razzak M.I., Anwar M.W., Akbar H. Arabic script based language character recognition: Nasta’liq vs. Naskh analysis. Proceedings of the 2013 World Congress on Computer and Information Technology (WCCIT).

[10] Naz S., Razzak M.I., Hayat K., Anwar M.W., Khan S.Z. (2014). Challenges in baseline detection of Arabic script based languages. Intelligent Systems for Science and Information: Extended and Selected Results from the Science and Information Conference 2013.

[11] Al Abodi J., Li X. (2014). An effective approach to offline Arabic handwriting recognition. Comput. Electr. Eng..

[12] Kacem A., Aouïti N., Belaïd A. Structural Features Extraction for Handwritten Arabic Personal Names Recognition. Proceedings of the 2012 International Conference on Frontiers in Handwriting Recognition.

[13] Sassoon R. (2003). Handwriting: The Way to Teach It.

[14] Azmi A.M., Alsaiari A. (2014). A calligraphic based scheme to justify Arabic text improving readability and comprehension. Comput. Hum. Behav..

[15] Adak C., Chaudhuri B.B., Blumenstein M. Legibility and aesthetic analysis of handwriting. Proceedings of the 2017 14th IAPR International Conference on Document Analysis and Recognition (ICDAR).

[16] Graham S., Weintraub N., Berninger V.W. (1998). The relationship between handwriting style and speed and legibility. J. Educ. Res..

[17] El-Sawy A., El-Bakry H., Loey M., Mastorakis N. (2016). An Intelligent Agent Tutor System for Detecting Arabic Children Handwriting Difficulty Based on Immediate Feedback. WSEAS Trans. Syst..

[18] Candela F., Romeo S., Faundez-Zanuy M., Ferrer-Ramos P. (2024). Cognitive Impairment Detection Based on Frontal Camera Scene While Performing Handwriting Tasks. Cogn. Comput..

[19] De Stefano C., Fontanella F., Impedovo D., Pirlo G., Scotto di Freca A. (2019). Handwriting analysis to support neurodegenerative diseases diagnosis: A review. Pattern Recognit. Lett..

[20] Balestrino M., Brugnolo A., Girtler N., Pardini M., Rizzetto C., Alì P.A., Cocito L., Schiavetti I. (2024). Cognitive impairment assessment through handwriting (COGITAT) score: A novel tool that predicts cognitive state from handwriting for forensic and clinical applications. Front. Psychol..

[21] Likforman-Sulem L., Esposito A., Faundez-Zanuy M., Clémençon S. (2015). Extracting style and emotion from handwriting. Advances in Neural Networks: Computational and Theoretical Issues.

[22] Ezz M., Sharaf M., Hassan A. (2019). Classification of Arabic writing styles in ancient Arabic manuscripts. Int. J. Adv. Comput. Sci. Appl..

[23] Adam K., Al-Maadeed S., Bouridane A. Letter-based classification of Arabic scripts style in ancient Arabic manuscripts: Preliminary results. Proceedings of the 2017 1st International Workshop on Arabic Script Analysis and Recognition (ASAR).

[24] Kaoudja Z., Khaldi B., Kherfi M.L. Arabic artistic script style identification using texture descriptors. Proceedings of the 2020 1st International Conference on Communications, Control Systems and Signal Processing (CCSSP).

[25] Khayyat M.M., Elrefaei L.A. (2020). A deep learning based prediction of arabic manuscripts handwriting style. Int. Arab J. Inf. Technol..

[26] Gürer D.Z., Gökbay İ.Z. (2024). Arabic Calligraphy Image Analysis with Transfer Learning. Electrica.

[27] Ghosh T., Sen S., Obaidullah S., Santosh K., Roy K., Pal U. (2022). Advances in online handwritten recognition in the last decades. Comput. Sci. Rev..

[28] Mezghani N., Mitiche A., Cheriet M. On-line recognition of handwritten arabic characters using a kohonen neural network. Proceedings of the Proceedings Eighth International Workshop on Frontiers in Handwriting Recognition.

[29] Tlemsani R., Belbachir K. An improved Arabic on-line characters recognition system. Proceedings of the 2018 International Arab Conference on Information Technology (ACIT).

[30] Alemayoh T.T., Shintani M., Lee J.H., Okamoto S. (2022). Deep-learning-based character recognition from handwriting motion data captured using IMU and force sensors. Sensors.

[31] Smitshuijzen E. (2009). Arabic Font Specimen Book.

[32] Janbi J. (2016). Classifying Arabic Fonts Based on Design Characteristics: PANOSE-A. Ph.D. Thesis.

[33] Lea C., Vidal R., Reiter A., Hager G.D., Hua G., Jégou H. (2016). Temporal Convolutional Networks: A Unified Approach to Action Segmentation. Proceedings of the Computer Vision—ECCV 2016 Workshops.

[34] Dai R., Minciullo L., Garattoni L., Francesca G., Bremond F. Self-Attention Temporal Convolutional Network for Long-Term Daily Living Activity Detection. Proceedings of the 2019 16th IEEE International Conference on Advanced Video and Signal Based Surveillance (AVSS).

[35] Zhang K., Zuo W., Chen Y., Meng D., Zhang L. (2017). Beyond a Gaussian Denoiser: Residual Learning of Deep CNN for Image Denoising. IEEE Trans. Image Process..

[36] Yan J., Mu L., Wang L., Ranjan R., Zomaya A. (2020). Temporal Convolutional Networks for the Advance Prediction of ENSO. Sci. Rep..

[37] Vaswani A., Shazeer N., Parmar N., Uszkoreit J., Jones L., Gomez A.N., Kaiser Ł., Polosukhin I. (2017). Attention is all you need. Adv. Neural Inf. Process. Syst..

[38] Bu S.J., Cho S.B. (2020). Time series forecasting with multi-headed attention-based deep learning for residential energy consumption. Energies.

[39] Zeiler M.D. (2012). Adadelta: An adaptive learning rate method. arXiv.

[40] Shapley L. (1953). A value for n-persons games. Ann. Math. Stud..

[41] Lundberg S.M., Lee S.I., Guyon I., Luxburg U.V., Bengio S., Wallach H., Fergus R., Vishwanathan S., Garnett R. (2017). A Unified Approach to Interpreting Model Predictions. Advances in Neural Information Processing Systems 30.

[42] Fryer D., Strümke I., Nguyen H. (2021). Shapley values for feature selection: The good, the bad, and the axioms. arXiv.

[43] Harris T.L., Rarick G.L. (1957). The problem of pressure in handwriting. J. Exp. Educ..

